# Correction from Harvard

**Published:** 1904-03

**Authors:** Eugene H. Smith


### Domestic Correspondence.

### CORRECTION FROM HARVARD.

To the Editor:

Sir,—Will you kindly publish this letter and by so doing correct an error in your editorial entitled, "Will Dental Colleges reverse their Decision?" In this editorial, which appeared in the February issue of the International Dental Journal, you are made to say,—

"The vote, however, on the Harvard application refusing permission for that school to remain at three years, etc."

Harvard did not make application for permission to keep to the three years' course. Harvard simply stated her position, and the reason therefor, hoping that the Association of Dental Faculties might see the wisdom of first materially advancing the entrance requirements, and bringing all schools to three years of nine months each, before entering upon a desultory course of four years for students insufficiently trained for a professional education. The committee, of which you were chairman, appointed by the Association, failed utterly to understand the scope of our entrance requirements, and reported accordingly, and the resignation of the Harvard Dental School followed.

Very truly yours,

Eugene H. Smith.

